# Time spent outdoors as an intervention for myopia prevention and control in children: an overview of systematic reviews

**DOI:** 10.1111/opo.12945

**Published:** 2022-01-24

**Authors:** Rohit Dhakal, Rakhee Shah, Byki Huntjens, Pavan K Verkicharla, John G Lawrenson

**Affiliations:** ^1^ Myopia Research Lab Prof. Brien Holden Eye Research Centre L V Prasad Eye Institute Hyderabad India; ^2^ Centre for Applied Vision Research School of Health Sciences, City University of London London UK

**Keywords:** children, intervention, light exposure, Myopia, outdoor time, overview

## Abstract

**Purpose:**

Outdoor light exposure is considered a safe and effective strategy to reduce myopia development and aligns with existing public health initiatives to promote healthier lifestyles in children. However, it is unclear whether this strategy reduces myopia progression in eyes that are already myopic. This study aims to conduct an overview of systematic reviews (SRs) reporting time spent outdoors as a strategy to prevent myopia or slow its progression in children.

**Methods:**

We searched the Cochrane Library, EMBASE, MEDLINE and CINAHL from inception to 1 November 2020 to identify SRs that evaluated the association between outdoor light exposure and myopia development or progression in children. Outcomes included incident myopia, prevalent myopia and change in spherical equivalent refraction (SER) and axial length (AL) to evaluate annual rates of myopia progression. The methodological quality and risk of bias of included SRs were assessed using the AMSTAR‐2 and ROBIS tools, respectively.

**Results:**

Seven SRs were identified, which included data from 47 primary studies with 63,920 participants. Pooled estimates (risk or odds ratios) consistently demonstrated that time outdoors was associated with a reduction in prevalence and incidence of myopia. In terms of slowing progression in eyes that were already myopic, the reported annual reductions in SER and AL from baseline were small (0.13–0.17 D) and regarded as clinically insignificant. Methodological quality assessment using AMSTAR‐2 found that all reviews had one or more critical flaws and the ROBIS tool identified a low risk of bias in only two of the included SRs.

**Conclusion:**

This overview found that increased exposure to outdoor light reduces myopia development. However, based on annual change in SER and AL, there is insufficient evidence for a clinically significant effect on myopia progression. The poor methodological quality and inconsistent reporting of the included systematic reviews reduce confidence in the estimates of effect.

**Key points**

- Overviews of systematic reviews (SRs) provide a comprehensive evidence map in a particular subject area.
- Pooled effect estimates from SRs demonstrate that increased time outdoors is associated with a reduction in myopia development, with small and clinically insignificant reductions in refractive error and axial length in eyes that are already myopic.
- Outdoor light exposure remains a safe and effective strategy to reduce myopia development and aligns with existing public health initiatives to promote healthier lifestyles in children.

## INTRODUCTION

Myopia is a complex ocular refractive condition with a multifactorial aetiology.^1^, ^2^ The rapid worldwide rise in the prevalence of myopia in the last two to three decades suggests a strong link between environmental factors and development of myopia.^3^ Various animal and human studies have reported that exposure to outdoor ambient light plays a protective role in myopia development.^4^, ^5^, ^6^, ^7^, ^8^, ^9^ While recent publications have reported that increasing time spent outdoors might also have an impact on myopia progression, there is some uncertainty regarding its inhibitory role on axial elongation.^6^, ^9^, ^10^ Although a number of hypotheses have been proposed for the protective effect of outdoor light including triggering dopamine release from the retina,^11^, ^12^ decreased pupil size due to high illumination thereby increasing the depth of focus and reducing retinal image blur^13^, ^14^ and exposure to shorter wavelengths of light,^15^, ^16^, ^17^, ^18^, ^19^, ^20^, ^21^ the precise mechanism still remains unclear.

Overviews of systematic reviews (SRs) aim to systematically retrieve and summarise the results of multiple SRs to provide a comprehensive evidence map in a particular subject area. An initial scoping of the literature on the role of time outdoors and myopia identified at least five recently published SRs.^22^, ^23^, ^24^, ^25^, ^26^ The objective of this overview is to synthesise and summarise the evidence on efficacy of outdoor light exposure on myopia prevalence, incidence and progression.

## METHODS

This overview was conducted in accordance with the criteria for conducting overviews of SRs described in Chapter 5 of the Cochrane Handbook for Systematic Reviews of Interventions.^27^ Prior to the start of the review process, the protocol was registered with the International prospective register of systematic reviews (PROSPERO registration number CRD42020214523).^28^

### Search methods for identification of reviews

We searched the following electronic databases: Cochrane Database of Systematic Reviews (CDSR), Database of Abstracts of Reviews of Effects (DARE), EMBASE, MEDLINE and CINAHL for relevant SRs. No date or language restriction was incorporated in the search strategy. The last date of search was 1 November, 2020. The search was supplemented by scanning the reference list of included reviews. To identify any ongoing SRs, protocols were searched in SRs registries, such as CDSR and PROSPERO. The search strategy for MEDLINE Central is provided in Appendix 1, and an individual search strategy for each of the databases is provided in File S1.

### Selection of reviews and assessment of eligibility criteria

Two reviewers (RD and RS) independently screened the titles and abstracts to identify potentially relevant studies for full text review. The full text of potentially relevant studies was assessed independently by the same reviewers to determine if they met the inclusion criteria. The inclusion criteria were: (i) any SRs (with or without meta‐analysis) that had evaluated the association between outdoor light exposure and myopia incidence, prevalence or progression in children; (ii) outdoor light exposure quantified either in the form of illuminance level (measured as lux) or total time spent outdoors (measured as number of exposure hours per day or per week) and (iii) age of participants ≤18 years, with or without myopia at baseline. Reviews were not excluded based on the type/form of interventions assigned to the participants for outdoor light exposure, nor the design of included studies e.g., cross‐sectional, cohort or randomised controlled trials. If a review included both children and adults, the required data from children only were extracted. Pairs of review authors (RD and BH, or RD and RS) independently extracted data from the included reviews on a previously piloted data extraction form. These data are represented in Table 1. The criteria for defining a systematic review were adopted from Martinic et al.^29^ Discrepancies were resolved through discussion between the two reviewers and when required, a third reviewer was consulted (JGL).

**TABLE 1:** Characteristics of included systematic reviews in the overviews

Review (Study design)	Number and name of databases searched (last date of search)	No. of primary studies included & type of study design	Definition of myopia	Total population included in review	Ethnicity & age groups (years)	Details of intervention	Review outcomes
Sherwin et al.^23^ (SR & MA)	4, MEDLINE, Web of Science, EMBASE and CENTRAL (Sept 2011)	23; 15 CS, 7 cohort and 1 RCT. MA was performed only in 7 CS	≤−0.50 D	23,739	Caucasian, Turkish, Asian; 0.5–20	**CS‐** Duration of outdoor exposure was recorded through questionnaires. Definition of exposure were sports, outdoor and sports, & only outdoor sports.	**Quantitative**‐ Myopia prevalence; **Narrative**‐ Myopia prevalence, myopia incidence and change in SER
Xiong et al.^24^ (SR & MA)	3, PubMed, EMBASE and Cochrane library (Dec 2015)	51; 4 CT, 17 cohort studies, and 30 CS. MA was performed in 25 studies	NR	72,327	Chinese, Caucasian, Indian, Malays, Mongolian, East Asian, African‐American, Turkish; 4–79	**CT‐** 40 min additional outdoor activities during school days vs. control; additional 40 min ROC programme vs. control; 80 min ROC programme vs. control and >14 h/week outdoor activities vs. control. **Cohort studies‐** Duration of outdoor exposure were recorded through interview or questionnaires administered to parents. **CS‐** Duration of outdoor exposure was recorded through questionnaires administered either to participants or parents	**Quantitative**‐ Myopia prevalence, myopia incidence, and change in SER
Deng and Pang^26^ (SR & MA)	6, Scopus, MEDLINE, EMBASE, VisionCite, PubMed and Cochrane library (March 2016)	5; 2 RCT, 2 cluster RCT and 1 NR about randomization	NR	3,272	Chinese; 6–18	**CT**‐ 40 min additional outdoor activities during school days vs. control; 80 min ROC programme vs. control; ROC programme vs. control; additional 40 min ROC programme vs. control and >14 h/week outdoor activities vs. control	**Quantitative**‐ Myopia incidence, change SER, change in AL
Ho et al.^22^ (SR & MA)	6, Cochrane Library, MEDLINE, CINAHL, PubMed, China Academic Journals full‐text database and National Digital Library of Theses and Dissertations in Taiwan (2019)	13; 6 CT, 3 cohort studies, and 4 CS	NR	15,081	Chinese, East Asian, Hispanic, Caucasian; 4–14	**CT‐** 7 h/week of outdoor exposure including recess and physical education vs. control; 7 h/week of exposure and extra 5 h/week after school vs. control; 80 min ROC programme vs. no special programme; ROC programme for 200 min/5 days vs. control; 40 min additional outdoor activities during school days vs. control; 2 additional 20 min recess programmes vs. control and >14–15 h/week of outdoor activities vs. control **Cohort studies‐** Outdoor light exposure was recorded through questionnaires **CS‐** Outdoor light exposure was recorded through questionnaires	**Quantitative**‐ Myopia prevalence, myopia incidence, change in SER and change in AL
Cao et al.^25^ (SR & MA)	5, PubMed, Science Direct, Cochrane Library, Chinese National Knowledge Infrastructure and Wanfang (Oct 2018)	5; 1 RCT, 4 cluster RCT	<−0.50 D	3,014	Chinese; 6–12	**CT‐** >14–15 h/week of outdoor activities vs. control; 2–3 h outdoor hikes/sports/activities on weekends at least twice/month vs. control; additional 40‐min outdoor activity in school days vs. control; 2 additional recess programmes of 20 min each in school days vs. control and ≥11 h of outdoor activities in 7 days vs. control	**Quantitative**‐ Myopia incidence, change in SER and change in AL
Anandita and Barliana^37^ (Narrative review)	1, MEDLINE (NR)	13; 8 CS, 5 Cohort	Ranged from <−0.50 to ≤−1.00 D	29,301	Chinese, East Asian, African, Caucasian, Arabian, Hispanic; 0–18	**Cohort studies‐** Outdoor light exposure was recorded through questionnaires **CS‐** Outdoor light exposure was recorded through questionnaires	**Narrative**: Myopia prevalence and myopia incidence
Eppenberger and Sturm^36^ (Narrative review)	2, PubMed and Cochrane Library (Jan 2019)	12; 3 CT, 7 cohort and 2 CS	Ranged from ≤−0.50 to ≤−1.00 D	32,381	Chinese, Malay, East Asian, Indian, African, Hispanic; 6–18	**CT‐** Additional 40 min of outdoor activities on each school day vs. control; 2 additional 20 min of ROC programme vs. control; ≥11 h of outdoor activities in 7 days vs. control **Cohort studies‐** Outdoor light exposure was recorded through questionnaires **CS‐** Outdoor light exposure was recorded through questionnaires	**Narrative**: Myopia prevalence, myopia incidence and change in SER

Abbreviations: CS, Cross‐sectional; CT, Clinical trial; MA, Meta‐analysis; NR, Not reported; RCT, Randomised clinical trial; ROC, Recess outside classroom; SER, Spherical equivalent refraction; SR, Systematic review; TSO, Time spent outdoors.

### Types of outcome measures

#### Primary outcomes

1. Efficacy of outdoor light exposure to prevent myopia onset measured as the number of cases of incident myopia for each year of follow‐up.
2. Efficacy of outdoor light exposure to control myopia progression measured as change in spherical equivalent refraction (SER) for each year of follow‐up.

#### Secondary outcomes

1. Association between outdoor light exposure and myopia prevalence.
2. Efficacy of outdoor light exposure to control myopia progression measured as change in axial length (AL) for each year of follow up.

### Quality and risk of bias assessment

The assessment of methodological quality and risk of bias (ROB) of the included SRs were conducted by pairs of review authors (RD and JGL, or RD and PKV). The methodological quality was assessed according to the criteria specified in the ‘A Measurement Tool to Assess Systematic Reviews 2’ (AMSTAR‐2) tool, ^30^ and risk of bias was assessed using the ‘Risk of Bias in Systematic Reviews’ (ROBIS) tool.^31^ Discrepancies between the two authors were resolved through discussion, or when required, a third reviewer was consulted (PKV or JGL).

### Data synthesis, presentation and analysis

A PRISMA (Preferred Reporting Items for Systematic Reviews and Meta‐analysis) 2020 flow diagram^32^ was used to summarise the selection of SRs. The characteristics of included and excluded SRs are tabulated descriptively and presented in Table 1. The outcome of AMSTAR‐2 and ROBIS assessments were represented in tabular and graphical format, respectively. Quantitative outcome data i.e., pooled estimates of primary and secondary outcome measures were presented as they were reported in the SRs i.e., odds ratio (OR) with 95% confidence interval (CI) for myopia prevalence, odds ratio or risk ratio (RR) with 95% CI for myopia incidence, mean difference (MD) with 95% CI for change in SER and AL. Forest plots were used to compare pooled estimates graphically, constructed using Review Manager (RevMan) software version 5.4.1 (The Cochrane Collaboration, revman.cochrane.org).^33^

For reviews reporting myopia incidence as an OR, the OR was converted to a RR using the formula described by Zhang and Yu.^34^ Continuous outcome data, i.e., change in SER and AL, were summarised as MD with 95% CI in the forest plot. The outcome measures for change in SER and AL were standardised to 1‐year time duration. One of the reviews reported a pooled estimate of change in SER over a period of 3 years. This was standardised to 1 year by dividing the overall MD by 2.3, because this review multiplied 1‐year follow‐up data from the primary studies by 2.3 to convert to 3‐years follow‐up data, considering reduction in myopia progression with age.^24^

A citation matrix was created to demonstrate the amount of overlap of primary studies between the included systematic reviews. The Corrected Covered Area (CCA) was calculated to provide a quantitative measure of the extent of overlap in the primary studies.^35^ CCA was calculated using the formula: CCA = (N−r)/(rc−r), where N = sum of total primary studies included in all the reviews, r = number of rows containing unique primary studies and c = number of columns, i.e., number of reviews.

## RESULTS

### Search results

**FIGURE 1:**
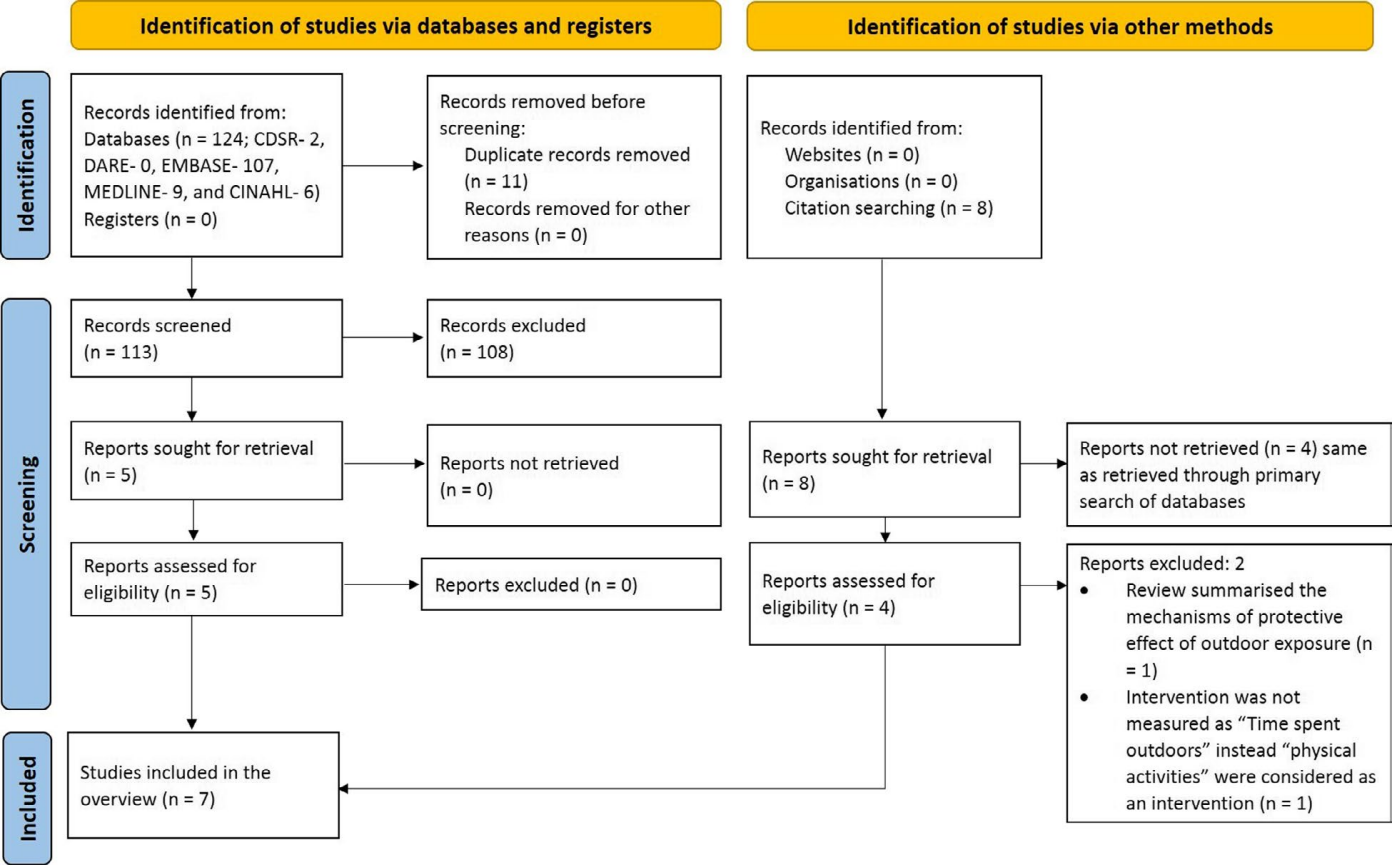
Preferred Reporting Items for Systematic Reviews and Meta‐Analyses (PRISMA) flow diagram presenting the process of study identification, screening and selection of systematic reviews

Bibliographic database searches identified 124 studies, of which 11 were duplicates. After screening titles and abstracts, 108 studies were excluded, and the full text of the remaining five studies were assessed for eligibility. All five SRs were included in this overview.^22^, ^23^, ^24^, ^26^, ^36^ Reference list scanning of included reviews identified four additional records that were assessed for eligibility,^25^, ^37^, ^38^, ^39^of which two were included.^25^, ^37^ Thus, a total of seven studies were included in the overview (Figure 1).

### Characteristics of included reviews

Table 1 summarises the characteristics of the included SRs. The total number of participants included in the reviews was 179,115 (63,920 unique participants), of which 117,433 (40,981 unique participants) were included in the quantitative synthesis. The definition of myopia was reported in four SRs, ranging from ≤−0.50 to ≤−1.00 D.^23^, ^25^, ^36^, ^37^ The majority of participants were younger than 18 years, although two reviews included older adults.^23^, ^24^ The included population were Caucasian, Turkish, East Asian, Chinese, Indian, Malay, Mongolian, African‐American and Hispanic.^22^, ^23^, ^24^, ^25^, ^26^, ^36^, ^37^ The details of interventions and reported outcomes are provided in Table 1. The characteristics of excluded SRs along with the justification for exclusion are detailed in the File S2.

### Risk of bias assessment

Table 2 summarises the methodological quality of included SRs, assessed using AMSTAR‐2. None of the SRs had registered or published a protocol prior to conducting the review, nor reported a list of excluded studies. Four SRs formally conducted a ROB assessment of included studies using a validated tool, one partially assessed ROB, and two SRs did not conduct an assessment of bias. Other methodological limitations included the lack of a comprehensive search strategy and poor justification for included study designs; three SRs failed to perform data extraction in duplicate.

**TABLE 2:** Methodological quality of the included systematic reviews as judged by the AMSTAR‐2 instrument

Items	Deng and Pang^26^	Sherwin et al.^23^	Anandita and Barliana^37^	Xiong et al.^24^	Ho et al.^22^	Cao et al.^25^	Eppenberger and Sturm^36^
1. Did the research questions and inclusion criteria for the review include the components of PICO?							
2. Did the report of the review contain an explicit statement that the review methods were established prior to conduct of the review and did the report justify any significant deviations from the protocol?							
3. Did the review authors explain their selection of the study designs for inclusion in the review?							
4. Did the review authors use a comprehensive literature search strategy?							
5. Did the review authors perform study selection in duplicate?							
6. Did the review authors perform data extraction in duplicate?							
7. Did the review authors provide a list of excluded studies and justify the exclusions?							
8. Did the review authors describe the included studies in adequate detail?							
9. Did the review authors use a satisfactory technique for assessing the risk of bias (ROB) in individual studies that were included in the review?							
10. Did the review authors report on the sources of funding for the studies included in the review?							
11. If meta‐analysis was justified, did the review authors use appropriate methods for statistical combination of results?							
12. If meta‐analysis was performed, did the review authors assess the potential impact of ROB in individual studies on the results of the meta‐analysis or other evidence synthesis?							
13. Did the review authors account for ROB in individual studies when interpreting/ discussing the results of the review?							
14. Did the review authors provide a satisfactory explanation for, and discussion of, any heterogeneity observed in the results of the review?							
15. If they performed quantitative synthesis, did the review authors carry out an adequate investigation of publication bias (small study bias) and discuss its likely impact on the results of the review?							
16. Did the review authors report any potential sources of conflict of interest, including any funding they received for conducting the review?							

Abbeviations: PICO, Patient Intervention Comparator Outcom.

Note: Colour coding indicates whether the study satisfied each AMSTAR‐2 item. Red = no; Green = yes; Blue = partially yes, and Yellow = not applicable. The grey shaded items represent critically important domains.

**FIGURE 2:**
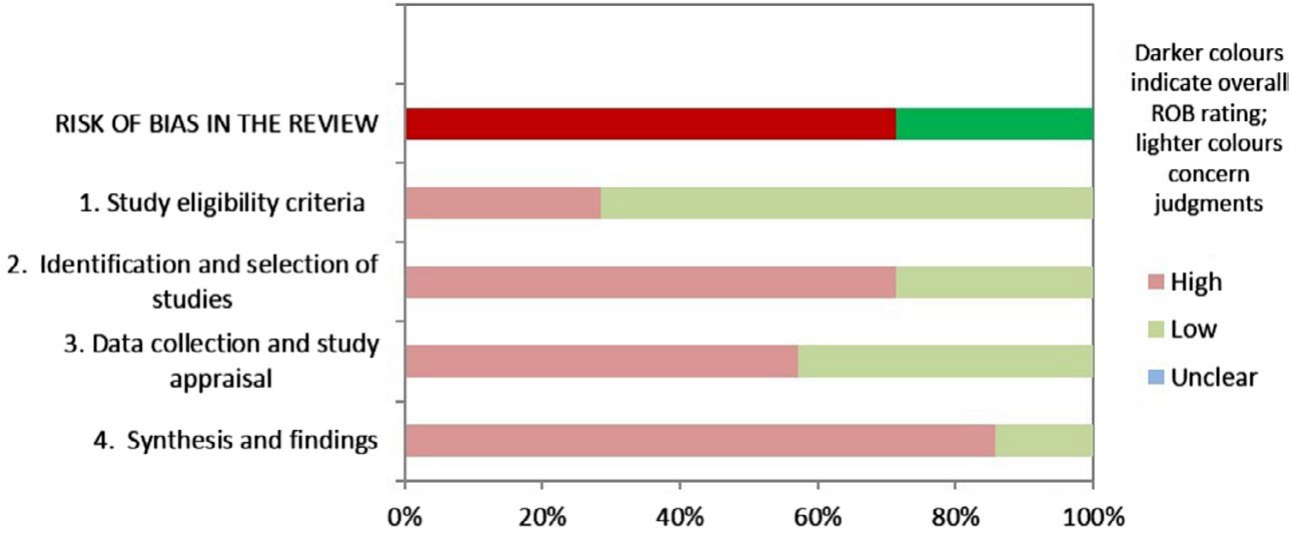
Graphical representation of Risk of Bias Assessment Tool for Systematic Reviews (ROBIS) assessment in seven included systematic reviews

Figure 2 shows the results of the ROBIS assessment. The ROBIS tool is divided into four domains. For Domain 1 (Study eligibility criteria), two reviews^36^, ^37^ were rated as ‘high concern’ based on restricted eligibility criteria of the included studies. For Domain 2 (Identification and selection of studies), five SRs^24^, ^25^, ^26^, ^36^, ^37^ were judged to be of high concern due to lack of a comprehensive search strategy or imposing language restrictions (studies published in English only). Furthermore, two SRs had no information on the number of authors involved in the identification and selection of the primary studies.^36^, ^37^ With regards to Domain 3 (Data collection and study appraisal), four SRs^23^, ^26^, ^36^, ^37^ were judged to be of high concern due to issues regarding the methods used to collect data and appraise studies (no formal ROB assessment was undertaken in two SRs). In Domain 4 (Synthesis and findings), high concerns were identified in four out of five reviews. The major concern in two reviews^22^, ^26^ was inappropriately combining results from different study designs. Two reviews^24^, ^37^ did not include results from all of the included primary studies to pool final estimates and the reason for excluding them was not reported. The ROBIS assessment for each of the included SRs is available in File S3.

### Overlapping of primary studies

The citation matrix showing the overlap of primary studies among seven SRs is presented in File S4. Using the formula mentioned in the methods section, we found CCA of 17.7%. Guidelines for interpreting CCA^35^ suggest that values greater than or equal to 15% indicate high overlap.

### Efficacy of outdoor light exposure

#### Myopia prevalence

**FIGURE 3:**
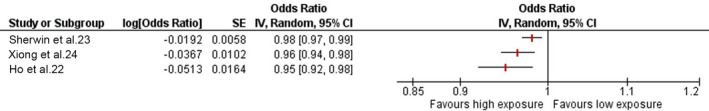
Forest plot showing pooled estimates of association between outdoor light exposure and myopia prevalence

Three out of the seven SRs synthesising data from a total of 24,889 unique participants reported the association between outdoor light exposure and myopia prevalence (Table 3 and Figure 3).^22^, ^23^, ^24^ All the reviews found a weak association between outdoor light exposure and myopia prevalence with an overall 2%–5% reduction in the odds of developing myopia for each additional hour spent outdoors per week.

**TABLE 3:** Myopia prevalence reported in included systematic reviews and meta‐analyses

Review study	Number of subjects (Number and design of primary studies)	Duration of effect (years)	Measure of effect (95% CI)	Direction of effect
Sherwin et al.^23^	9,885 (7, CS)	NA	OR 0.98 (0.97, 0.99)	Favours high outdoor exposure
Xiong et al.^24^	23,112 (13, CS)	NA	OR 0.96 (0.94, 0.98)	Favours high outdoor exposure
Ho et al.^22^	5,745 (4, CS)	NA	OR 0.95 (0.92, 0.99)	Favours high outdoor exposure

Abbreviations: CI, Confidence interval; CS, Cross‐sectional studies; NA, Not applicable; OR, Odds ratio.

#### Myopia incidence

**FIGURE 4:**
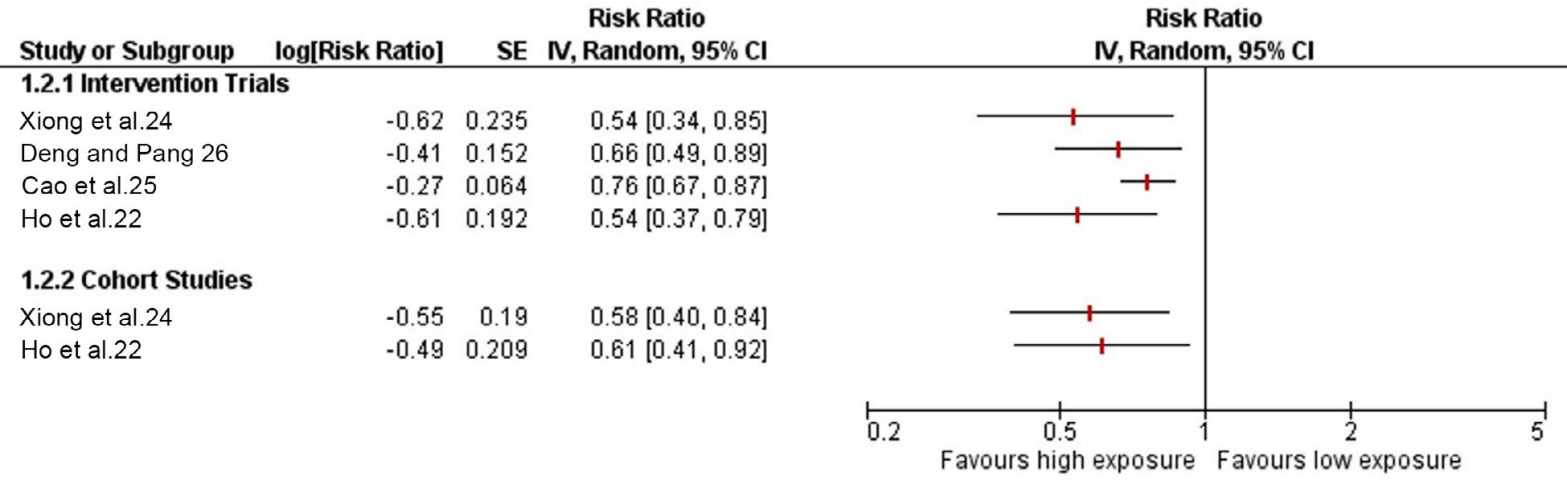
Forest plot showing pooled estimates of myopia incidence over a period of 1 year from baseline. *Reported change in 3 years

Four out of the seven SRs synthesising data from a total of 7783 unique participants (3161 participants from clinical trials and 4622 participants from cohort studies) reported the association between outdoor light exposure and myopia incidence (Table 4 and Figure 4). Two of the reviews reported outcomes over a period of 1 year,^22^, ^26^ one review reported outcomes over a period of 3 years^24^ and one did not report the duration over which the effect was observed.^25^ Likewise, one of the four reviews reported measure of outcome as OR, whilst the others reported as RR with 95% CI.

**TABLE 4:** Myopia incidence reported in included systematic reviews and meta‐analyses

Review study	Number of subjects (Number and design of primary studies)	Duration of effect (years)	Measure of effect (95% CI)	Measure of effect standardised to RR (95% CI)	Direction of effect
Xiong et al.^24^	2,865 (3, CT)	3	RR 0.54 (0.34, 0.85)	RR 0.54 (0.34, 0.85)	Favours high outdoor exposure
Deng and Pang^26^	2,885 (4, CT)	1	RR 0.66 (0.49, 0.89)	RR 0.66 (0.49, 0.89)	Favours high outdoor exposure
Cao et al.^25^	2,590 (3, CT)	NR	RR 0.76 (0.67, 0.87)	RR 0.76 (0.67, 0.87)	Favours high outdoor exposure
Ho et al.^22^	4,714 (5, CT)	1	OR 0.50 (0.37, 0.69)	RR 0.54 (0.37, 0.79)	Favours high outdoor exposure
Xiong et al.^24^	4,064 (2, Cohort)	3	RR 0.57 (0.40, 0.83)	RR 0.57 (0.40, 0.83)	Favours high outdoor exposure
Ho et al.^22^	4,622 (3, Cohort)	1	OR 0.57 (0.35, 0.92)	RR 0.61 (0.41, 0.92)	Favours high outdoor exposure

Abbreviations: CI, Confidence interval; CT, Clinical trial; NR, Not reported; OR, Odds ratio; RR, Risk ratio.

All four SRs reported a significantly reduced risk of myopia development in children with increased hours of outdoor activities (Table 4 and Figure 4). Of the SRs which included only clinical trials, the risk of developing myopia from baseline ranged from 0.54 to 0.76. The estimates from cohort studies were similar to that of intervention studies.

#### Change in myopic refractive error

**FIGURE 5:**
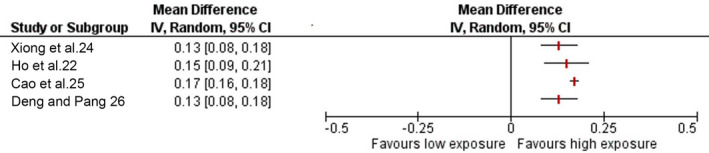
Forest plot showing pooled estimates of change in spherical equivalent refraction (SER) from baseline (1 year). *Did not report duration over which change was assessed

Four out of seven SRs synthesising data from a total of 4406 unique participants reported the effect of outdoor light exposure against change in SER (Table 5 and Figure 5). Two reviews reported the change over a period of 1 year,^22^, ^26^ one over a period of 3 years^24^ and one did not report the duration of effect.^25^ All included SRs reported lower rates of myopia progression in the higher light exposure group with annual mean reduction in progression ranging between 0.13 to 0.17 D.

**TABLE 5:** Change in spherical equivalent refractive error reported in included systematic reviews and meta‐analyses

Review study	Number of subjects (Number and design of primary studies)	Duration of effect (years)	Measure of effect (D), MD (95% CI)	Measure of effect standardised to 1 year (D) MD (95% CI)	Direction of effect
Xiong et al.^24^	2,865 (3, CT)	3	0.30 (0.18, 0.41)	0.13 (0.08, 0.18)	Favours high outdoor exposure
Ho et al.^22^	4,406 (6, CT)	1	0.15 (0.09, 0.22)	0.15 (0.09, 0.22)	Favours high outdoor exposure
Cao et al.^25^	2,729 (4, CT)	NI	0.17 (0.16, 0.18)	0.17 (0.16, 0.18)	Favours high outdoor exposure
Deng and Pang^26^	3,272 (5, CT)	1	0.13 (0.08, 0.18)	0.13 (0.08, 0.18)	Favours high outdoor exposure

Abbreviations: CI, Confidence interval; CT, Clinical trials; D, Dioptres; MD, Mean difference.

#### Change in axial length

**FIGURE 6:**
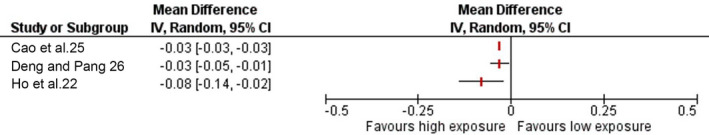
Forest plot showing pooled estimates of change in axial length from baseline (1 year). *Did not report duration over which change was assessed

Three of the seven SRs synthesising data from a total of 3,903 unique participants reported the effect of high outdoor light exposure against change in axial length (Table 6 and Figure 6).^22^, ^25^, ^26^ The forest plot in Figure 6 shows the direction of effect for all of the three SRs. The pooled MD change over a period of 1 year from baseline ranged from −0.03 mm to −0.08 mm.

**TABLE 6:** Change in axial length reported in included systematic reviews and meta‐analyses

Review study	Number of subjects (Number and design of primary studies)	Duration of effect (years)	Measure of effect (mm) (95% CI)	Direction of effect
Deng and Pang^26^	2,658 (3, CT)	1	MD −0.03 (−0.05, 0.00)	Favours high outdoor exposure
Cao et al.^25^	2,658 (3, CT)	NI	MD −0.03 (−0.03, −0.03)	Favours high outdoor exposure
Ho et al.^22^	3,903 (4, CT)	1	MD −0.08 (−0.14, −0.02)	Favours high outdoor exposure

Abbreviations: CI, Confidence interval; CT, Clinical trials; MD, Mean difference.

The conclusions of the two narrative reviews were consistent with those having a quantitative synthesis. Eppenberger and Sturm critically analysed the role of time exposed to outdoor light and concluded that increased outdoor light exposure could potentially lower the rate of myopia prevalence and incidence, as well as slow its progression.^36^ Likewise, Anadita and Barliana also reported the protective effect of outdoor light exposure against myopia incidence.^37^

## DISCUSSION

This overview aimed to bring together, appraise and synthesise the results of related systematic reviews that evaluated the relationship between outdoor light exposure and myopia. The overview summarised the results of seven SRs that included data from 47 unique primary studies published between 1977 and 2018, and synthesising data from 63,920 participants. The cumulative evidence suggests that exposure to higher outdoor light levels is associated with a reduction of myopia prevalence and incidence in children and provides equivocal evidence for a reduction in myopia progression (based on change in SER and axial length) in those who were already myopic.

The earlier the onset of myopia, the greater the likelihood of high myopia later in life,^40^, ^41^ increasing the risk of developing myopia related pathology,^42^, ^43^, ^44^ with a reduction in quality of life^45^ and an associated economic burden.^46^, ^47^ Each dioptre progression of myopia is shown to increase the likelihood of developing myopic maculopathy by 40% irrespective of the degree of myopic refractive error.^48^ Although the sight‐threatening pathologies associated with myopia usually occur later in life, the underlying myopia develops during childhood, and therefore interventions to prevent or reduce the progression of myopia need to be delivered during this period. Increased exposure to outdoor light is the safest, most cost‐effective and non‐invasive intervention to prevent or delay the onset of myopia.

Three SRs investigated the relationship between outdoor light exposure and myopia prevalence and reported pooled estimates from a meta‐analysis of cross‐sectional studies.^22^, ^23^, ^24^ These reviews reported effect sizes ranging from a 2%–5% reduction in the odds of developing myopia for each additional hour spent outdoors per week. Estimates of incident myopia were reported in four reviews.^22^, ^24^, ^25^, ^26^ The pooled effect sizes from cohort and intervention studies were similar; exposure to higher level of outdoor light was associated with a 39%–43% relative risk reduction in incident myopia from cohort studies, and 24%–46% relative risk reduction from clinical trials.

In addition to the reported benefits of outdoor light exposure on preventing myopia, time spent outdoors also has other health benefits. The most recent report from International Myopia Institute concluded that ‘compared with other measures, spending more time outdoors is the safest strategy and aligns with other existing health initiatives, for example, obesity prevention, by promoting a healthier lifestyle for children and adolescents’.^49^ Several public health policies based on outdoor light exposure are already implemented in China, Taiwan, Singapore and other East Asian countries at school level to prevent or delay the onset of myopia.^6^, ^10^, ^50^, ^51^ Some interventions suggested in the literature include a minimum one hour of recess outside the classroom; classrooms with many large windows; increasing awareness among parents and school children on the importance of outdoor light exposure; community centres to organise outdoor games/programmes to motivate children's participation; concept of nature kindergartens and school excursions to outdoor areas.^52^, ^53^, ^54^

Whilst there is a consensus on the protective effect of outdoor light exposure on preventing the onset of myopia, its role in slowing progression in eyes that are already myopic remains controversial. Four SRs^22^, ^24^, ^25^, ^26^ provided pooled estimates of changes in SER and AL from prospective intervention trials. One review^24^ reported that additional time outdoors was ineffective in slowing myopia progression, based on a sub‐group analysis of SER in myopic subjects only and the lack of a dose‐response relationship. By contrast, three other reviews^22^, ^25^, ^26^ concluded that additional time spent outdoors helped to slow progression in terms of change in SER and AL. The magnitude of the annual reduction in SER ranged from 0.13 to 0.17 D, which is consistent with a reduction of 0.03 to 0.08 mm change in AL. A consensus workshop, Controlling the Progression of Myopia, sponsored by the United States Food and Drug Administration (FDA) and attended by experts in myopia from several professional organisations, concluded that a minimum difference in refractive error of 0.75 D between the treatment groups would be considered clinically significant over a period of 3 years (change of 0.25 D annually).^55^ This corresponds to an approximate change of 0.11 mm/year in axial length. Based on these minimal changes needed in SER or AL to claim clinical significance, the estimates of annual change in SER and AL reported in the included reviews are noticeably lower than 0.25 D and 0.11 mm, respectively.

We used two validated critical appraisal tools to evaluate the methodological quality and ROB of the included SRs in this overview. Five out of seven SRs were judged as “high concern” in ROBIS assessment. The major concerns were the lack of a registered or published protocol before conducting SR, language restriction during the search process, not conducting study selection and data extraction in duplicate and inappropriately combining results during meta‐analysis. Two SRs inappropriately combined results from different study designs.^25^, ^26^ The Cochrane Handbook for Systematic Reviews of Interventions recommends not to combine results from different study designs in view of high heterogeneity and different sources of bias that could lead to imprecise estimates.^56^ Three SRs did not mention involvement of two authors to independently duplicate the steps of review process.^23^, ^36^, ^37^ Involvement of two review authors working independently and the presence of a third author in the review process to resolve discrepancies is a vital step of a systematic review that helps in minimising errors in selecting studies, extracting data and conducting ROB.^57^ The limitations in the conduct of the SRs included in this overview need to be taken into account when considering the precision of the pooled estimates and how certain we can be that the effect estimates are adequate to support a recommendation.

A strength of the current overview is the methodology adopted to conduct the review process, which is consistent with best practice.^27^ A protocol was registered prior to conducting the overview, a robust search matrix was designed, multiple authors were involved in the review process and standard tools were used to assess the methodological quality and ROB of the reviews. Given that majority of these reviews included children younger than 18 years of age and of different ethnicities, the results of this overview are generalisable to most ethnic groups, with cautious application in context to an adult population above 18 years of age. There are several limitations of the evidence base that are worth highlighting. First, despite axial length being the most reliable and repeatable parameter to measure myopia progression, relatively few of the included clinical trials (*n* = 4) measured axial length. It is important that future longitudinal studies and clinical trials investigating efficacy of time spent outdoors in controlling myopia include axial length as well as refractive error as primary outcome measures. Second, many of the included studies used participant self‐reporting to obtain information about time spent outdoors from parents and/or children. Studies have identified a poor correlation between subjectively obtained versus objectively measured information on outdoor activity.^58^, ^59^ More recent observational studies and clinical trials have used light trackers to quantify the amount of light exposure and total duration of time spent outdoors objectively.^8^, ^9^, ^60^, ^61^, ^62^ Future trials should continue to use such objective measures to define light exposure more accurately. Third, the majority of studies investigated the role of outdoor light exposure in isolation. However, there are several other factors such as parental myopia, urbanisation, educational level, duration and distance of near work, which could interact and contribute to myopiogenesis.^63^, ^64^, ^65^ Interaction between near work and outdoor light exposure has been reported in the literature suggesting slower myopia progression in children who spent more time outdoors and less time performing near work and vice‐versa.^64^, ^66^ These complex behavioural variables act as confounders which need to be considered when investigating the causal relationship between environmental factors and myopia development.

There is a paucity of evidence concerning the efficacy of outdoor light exposure against myopia onset or progression in adults. Likewise, the dose‐response relationship between (a) the illuminance level and (b) the duration of exposure to outdoor light and myopia warrants further investigation. While the role of different wavelengths of light in myopiogenesis is being investigated through short‐term experimental designs in humans,^15^, ^67^ the potential role of wavelength of light in controlling myopia progression needs to be explored further through clinical trials.

In conclusion, the current overview of SRs provides evidence for the protective effect of outdoor light exposure against the development of incident myopia. Increased outdoor light exposure should therefore be recommended as a safe and effective strategy to reduce myopia development. Furthermore, the intervention aligns with existing public health initiatives to promote healthier lifestyles in children. However, the impact of time outdoors in slowing myopia progression in eyes that are already myopic remains uncertain. Poor methodological quality and inconsistent reporting of the included systematic reviews reduces the confidence in the estimates of effect. We recommend that systematic reviewers should consult PRISMA and AMSTAR when conducting and reporting systematic reviews. Furthermore, developers of clinical practice guidelines should consider methodological quality when guideline recommendations are underpinned by systematic reviews.

## CONFLICT OF INTEREST

The authors report no conflicts of interest and have no proprietary interest in any of the materials mentioned in this article.

## AUTHOR CONTRIBUTIONS

**Rohit Dhakal:** Data curation (equal); Formal analysis (lead); Methodology (lead); Project administration (lead); Writing – original draft (lead); Writing – review & editing (lead). **Rakhee Shah:** Data curation (equal); Methodology (equal); Writing – review & editing (equal). **Byki Huntjens:** Data curation (equal); Methodology (equal); Writing – review & editing (equal). **Pavan K Verkicharla:** Data curation (equal); Methodology (equal); Writing – review & editing (equal). **John G Lawrenson:** Conceptualization (lead); Data curation (equal); Methodology (equal); Supervision (lead); Writing – review & editing (equal).

## Supporting information

File S1

File S2

File S3

File S4

## APPENDIX 1

### Search strategy for medline central database

S1. (MH "Child") OR (MH "Child, Preschool") OR (MH "Adult") OR (MH "Adolescent")

S2. AB child* OR AB student* OR AB "school age*" OR AB "school children" OR AB juvenile OR AB kid* OR AB teen* OR AB adolescen* OR AB youth* OR AB preschool* OR AB adult* OR AB p#ediatric

S3. S1 OR S2

S4. AB "Time N3 outdoor*" OR AB "environment* N3 exposure*" OR AB outdoor* OR AB "physical activit*" OR AB "outdoor activiti*" OR AB outside OR AB natur* OR AB exercise* OR AB "play* N3 outdoor" OR AB "light N3 expos*" OR AB leisure OR AB sport* OR sunlight OR daylight OR daytime

S5. (MH "Myopia") OR (MH "Myopia, Degenerative")

S6. AB myopi* OR AB myope* OR AB nearsight* OR AB near‐sight* OR AB shortsight* OR AB short‐sight* OR AB "refractive error*" OR AB refracti*

S7. S5 OR S6

S8. (MH "Control")

S9. AB prevent* OR AB control OR AB prophyla* OR AB therapy OR AB intervention* OR AB strateg*

S10. S8 OR S9

S11. S7 AND S10

S12. (MH "Systematic Review") OR (MH "SRs as Topic")

S13. AB "systematic review" OR AB ("systematic review and meta‐analysis") OR AB "intervention review"

S14. S11 OR S12

S15. S3 AND S4 AND S7 AND S14

S16. S3 AND S4 AND S11 AND S14
