# Supplementary material for: Time spent outdoors as an intervention for myopia prevention and control in children: an overview of systematic reviews
Source: Ophthalmic Physiol Opt. 2022 Jan 24;42(3):545–58. doi: 10.1111/opo.12945 (PMC9305934; doi:10.1111/opo.12945)
Supplement: Supplementary file 1 — File S1 [file OPO-42-545-s004.docx]

**Search strategy for individual databases**

1. **Embase**

1 child/ or juvenile/ or preschool child/ or school child/

2 adult/ or adult child/ or young adult/

3 adolescent/

4 1 or 2 or 3

5 (child* or student* or "school age*" or "school children" or juvenile or kid* or teen* or

adolescen* or youth* or preschool* or adult* or p?ediatric*).ab.

6 4 or 5

7 ("Time ADJ3 outdoor*" or "environment* ADJ3 exposure*" or outdoor* or "physical

activit*" or "outdoor activit*" or outside or natur* or exercise* or "play* ADJ3 outdoor" or

"light ADJ3 expos*" or leisure or sport* or sunlight or daylight or daytime).ab.

8 high myopia/ or degenerative myopia/ or myopia/

9 (myopi* or myope* or nearsight* or near-sight* or shortsight* or short-sight* or "refractive

error*" or refracti*).ab.

10 8 or 9

11 "prevention and control"/ or control/

12 (prevent* or control* or prophyla* or therap* or intervention* or strateg*).ab.

13 11 or 12

14 "systematic review"/ or meta analysis/ or "review"/

15 ("systematic review" or "systematic review and meta-analysis" or "intervention

review").ab.

16 14 or 15

17 10 and 13

18 6 and 7 and 10 and 16

19 6 and 7 and 16 and 17

20 18 or 19

1. **CDSR (Cochrane database for systematic review)**

1. Child.mp. [mp=title, short title, abstract, full text, keywords, caption text]

2. juvenile.mp. [mp=title, short title, abstract, full text, keywords, caption text]

3. "preschool child".mp. [mp=title, short title, abstract, full text, keywords, caption text]

4. "school child".mp. [mp=title, short title, abstract, full text, keywords, caption text]

5. adult.mp. [mp=title, short title, abstract, full text, keywords, caption text]

6. "young adult".mp. [mp=title, short title, abstract, full text, keywords, caption text]

7. "adolescent".mp. [mp=title, short title, abstract, full text, keywords, caption text]

8. 1 or 2 or 3 or 4 or 5 or 6 or 7

9. "Time spent outdoors".mp. [mp=title, short title, abstract, full text, keywords, caption text]

10. "Outdoor exposure".mp. [mp=title, short title, abstract, full text, keywords, caption text]

11. "Time outdoor".mp. [mp=title, short title, abstract, full text, keywords, caption text]

12. outdoor.mp. [mp=title, short title, abstract, full text, keywords, caption text]

13. "light exposure".mp. [mp=title, short title, abstract, full text, keywords, caption text]

14. "daylight".mp. [mp=title, short title, abstract, full text, keywords, caption text]

15. "sunlight".mp. [mp=title, short title, abstract, full text, keywords, caption text]

16. "Outdoor light exposure".mp. [mp=title, short title, abstract, full text, keywords, caption

text]

17. 9 or 10 or 11 or 12 or 13 or 14 or 15 or 16

18. myopia.mp. [mp=title, short title, abstract, full text, keywords, caption text]

19. shortsightedness.mp. [mp=title, short title, abstract, full text, keywords, caption text]

20. nearsightedness.mp. [mp=title, short title, abstract, full text, keywords, caption text]

21. shortsight.mp. [mp=title, short title, abstract, full text, keywords, caption text]

22. nearsight.mp. [mp=title, short title, abstract, full text, keywords, caption text]

23. short-sight.mp. [mp=title, short title, abstract, full text, keywords, caption text]

24. near-sight.mp. [mp=title, short title, abstract, full text, keywords, caption text]

25. 18 or 19 or 20 or 21 or 22 or 23 or 24

26. "myopia control".mp. [mp=title, short title, abstract, full text, keywords, caption text]

27. "anti myopia strategy".mp. [mp=title, short title, abstract, full text, keywords, caption text]

28. "myopia intervention".mp. [mp=title, short title, abstract, full text, keywords, caption text]

29. 26 or 27 or 28

30. 8 and 17 and 25

31. limit 30 to full systematic reviews

1. **DARE (Database of abstract of reviews of effect)**

1. child.mp. [mp=title, full text, keywords]

2. juvenile.mp. [mp=title, full text, keywords]

3. "preschool child".mp. [mp=title, full text, keywords]

4. "school child".mp. [mp=title, full text, keywords]

5. adult.mp. [mp=title, full text, keywords]

6. "young adult".mp. [mp=title, full text, keywords]

7. "adolescent".mp. [mp=title, full text, keywords]

8. teen*.mp. [mp=title, full text, keywords]

9. 1 or 2 or 3 or 4 or 5 or 6 or 7 or 8

10. "Time spent outdoors".mp. [mp=title, full text, keywords]

11. "Outdoor exposure".mp. [mp=title, full text, keywords]

12. "Time outdoor".mp. [mp=title, full text, keywords]

13. outdoor.mp. [mp=title, full text, keywords]

14. "light exposure".mp. [mp=title, full text, keywords]

15. "daylight".mp. [mp=title, full text, keywords]

16. "sunlight".mp. [mp=title, full text, keywords]

17. "Outdoor light exposure".mp. [mp=title, full text, keywords]

18. 10 or 11 or 12 or 13 or 14 or 15 or 16 or 17

19. myopia.mp. [mp=title, full text, keywords]

20. shortsightedness.mp. [mp=title, full text, keywords]

21. nearsightedness.mp. [mp=title, full text, keywords]

22. shortsight.mp. [mp=title, full text, keywords]

23. nearsight.mp. [mp=title, full text, keywords]

24. short-sight.mp. [mp=title, full text, keywords]

25. near-sight.mp. [mp=title, full text, keywords]

26. 19 or 20 or 21 or 22 or 23 or 24 or 25

27. "myopia control".mp. [mp=title, full text, keywords]

28. "anti myopia strategy".mp. [mp=title, full text, keywords]

29. "myopia intervention".mp. [mp=title, full text, keywords]

30. (prevent* or intervent* or therap* or prophylactic* or control* or strateg*).af.

31. 27 or 28 or 29 or 30

33. 26 and 31

34. 9 and 18 and 33

1. **CINAHL**

S16- S3 AND S6 AND S11 AND S14

S15- S3 AND S6 AND S9 AND S14

S14- S12 OR S13

S13- AB "systematic review" OR AB ( "systematic review and meta-analysis" ) OR AB

"intervention review"

S12- (MH "Meta Analysis") OR (MH "Systematic Review")

S11- S9 AND S10

S10- AB prevent* OR AB control* OR AB prophyla* OR AB therap* OR AB intervention* OR AB

strateg*

S9- S7 OR S8

S8- AB myopi* OR AB myope* OR AB nearsight* OR AB near-sight* OR AB shortsight* OR AB

short-sight* OR AB "refractive error*" OR AB refracti*

S7- (MH "Myopia")

S6- S4 OR S5

S5- AB sunlight OR AB daylight OR AB daytime

S4- AB "time N3 outdoor*" OR AB "environment* N3 exposure*" OR AB outdoor* OR AB

"physical S4- activit*" OR AB "outdoor activit*" OR AB outside OR AB natur* OR AB exercise*

OR AB "play* N3 outdoor" OR AB "light N3 expos*" OR AB leisure OR AB sport*

S3- S1 OR S2

S2- AB child* OR AB student* OR AB "school age*" OR AB "school children" OR AB juvenile OR

AB kid* OR AB teen* OR AB adolescen* OR AB youth* OR AB preschool* OR AB adult* OR

AB p#ediatric*

S1- (MH "Child") OR (MH "Adolescence") OR (MH "Child, Preschool")

1. **Medline**

S1- (MH "Child") OR (MH "Child, Preschool") OR (MH "Adult") OR (MH "Adolescent")

S2- AB child* OR AB student* OR AB "school age*" OR AB "school children" OR AB juvenile OR

AB kid* OR AB teen* OR AB adolescen* OR AB youth* OR AB preschool* OR AB adult* OR AB

p#ediatric

S3- S1 OR S2

S4- AB "Time N3 outdoor*" OR AB "environment* N3 exposure*" OR AB outdoor* OR AB

"physical activit*" OR AB "outdoor activiti*" OR AB outside OR AB natur* OR AB exercise* OR

AB "play* N3 outdoor" OR AB "light N3 expos*" OR AB leisure OR AB sport* OR sunlight OR

daylight OR daytime

S5- (MH "Myopia") OR (MH "Myopia, Degenerative")

S6- AB myopi* OR AB myope* OR AB nearsight* OR AB near-sight* OR AB shortsight* OR AB

short-sight* OR AB "refractive error*" OR AB refracti*

S7- S5 OR S6

S8- (MH "Control")

S9- AB prevent* OR AB control OR AB prophyla* OR AB therapy OR AB intervention* OR AB

strateg*

S10- S8 OR S9

S11 S7 AND S10

S12- (MH "Systematic Review") OR (MH "Systematic Reviews as Topic")

S13- AB "systematic review" OR AB ( "systematic review and meta-analysis" ) OR AB

"intervention review"

S14- S11 OR S12

S15- S3 AND S4 AND S7 AND S14

S16- S3 AND S4 AND S11 AND S14
