# Supplementary material for: Time spent outdoors as an intervention for myopia prevention and control in children: an overview of systematic reviews
Source: Ophthalmic Physiol Opt. 2022 Jan 24;42(3):545–58. doi: 10.1111/opo.12945 (PMC9305934; doi:10.1111/opo.12945)
Supplement: Supplementary file 2 — File S2 [file OPO-42-545-s003.docx]

| Review (Study design) | Number and name of databases searched (last date of search) | No. of primary studies included & type of study design | Total population included in review | Ethnicity and age range in years | Details of intervention | Reason for exclusion |
| --- | --- | --- | --- | --- | --- | --- |
| Zhang 2019, (Narrative review) | NR | Not clear | NR | NR | Discussed the protective mechanisms of outdoor light exposure against myopia | Not a systematic review and summarized different possible mechanisms involved in protective effect of outdoor light exposure. Efficacy of outdoor light exposure was not considered as an intervention against myopia development or progression in this review |
| Thykjaer 2017  (SR) | 3, PubMed, MEDLINE, EMBASE (Feb 2015) | 9; 6 CS, 2 cohort and 1 case-control | 17634 | Caucasians, Jordanian, Singaporean; mostly 7-15 years, few were above 20 years | Physical activities | Physical activity was considered as an intervention instead of outdoor light exposure |

**List of Excluded studies**

**Table 1A.** Characteristics of excluded studies and reason for exclusion

Abbreviations: NR- Not reported, CS- Cross-sectional studies
