# Supplementary material for: Time spent outdoors as an intervention for myopia prevention and control in children: an overview of systematic reviews
Source: Ophthalmic Physiol Opt. 2022 Jan 24;42(3):545–58. doi: 10.1111/opo.12945 (PMC9305934; doi:10.1111/opo.12945)
Supplement: Supplementary file 3 — File S3 [file OPO-42-545-s001.docx]

**Figure 2A.** Risk of bias assessment of the included systematic reviews as assessed by ROBIS tool.

| **Review** | **Phase 2** | | | | | **Phase 3** |
| --- | --- | --- | --- | --- | --- | --- |
|  | **1. Study eligibility criteria** | | **2. Identification and selection of studies** | **3. Data collection and study appraisal** | **4. Synthesis and findings** | **Risk of bias in the review** |
| Sherwin 2012 | ☺ | ☺ | | ☹ | ☹ | ☹ |
| Anandita 2015 | ☹ | | ☹ | ☹ | ☹ | ☹ |
| Xiong 2017 | ☺ | | ☹ | ☺ | ☹ | ☹ |
| Deng 2019 | ☺ | | ☹ | ☹ | ☹ | ☹ |
| Ho 2019 | ☺ | | ☺ | ☺ | ☹ | ☺ |
| Cao 2019 | ☺ | | ☹ | ☺ | ☺ | ☺ |
| Eppenberger 2020 | ☹ | | ☹ | ☹ | ☹ | ☹ |

☺ indicates low risk and ☹ indicates high risk
