# Supplementary material for: Time spent outdoors as an intervention for myopia prevention and control in children: an overview of systematic reviews
Source: Ophthalmic Physiol Opt. 2022 Jan 24;42(3):545–58. doi: 10.1111/opo.12945 (PMC9305934; doi:10.1111/opo.12945)
Supplement: Supplementary file 4 — File S4 [file OPO-42-545-s002.docx]

**Citation matrix**

**Figure 3A.** Citation matrix of the included studies for each review

| Primary studies | Study design | Deng 2019 | Sherwin 2012 | Xiong 2017 | Ho 2019 | Cao 2019 | Anandita 2015* | Eppenberger 2020* | Total number of overlap in reviews (/7) |
| --- | --- | --- | --- | --- | --- | --- | --- | --- | --- |
| Deng 2010 | CS | - | + | + | - | - | + | - | 3 |
| Dirani 2009 | CS | - | + | + | - | - | - | + | 3 |
| Ip 2008 | CS | - | + | + | + | - | + | - | 4 |
| Khader 2006 | CS | - | + | - | - | - | + | - | 2 |
| Low 2010 | CS | - | + | + | - | - | + | - | 3 |
| Lu 2009 | CS | - | + | + | - | - | + | - | 3 |
| Mutti 2002 | CS | - | + | + | - | - | + | - | 3 |
| Wu 2010 | CS | - | + | - | - | - | + | - | 2 |
| Saw 2001 | CS | - | + | - | - | - | - | - | 1 |
| Tan 2000 | CS | - | + | - | - | - | - | - | 1 |
| Rose 2008 | CS | - | + | - | - | - | - | - | 1 |
| Zhang 2010 | CS | - | + | - | - | - | - | - | 1 |
| Saw 2002 | CS | - | + | - | - | - | - | - | 1 |
| Rose 2008 | CS | - | + | - | - | - | - | - | 1 |
| MA 2010 | CS | - | + | - | - | - | - | - | 1 |
| Chua 2015 | CS | - | - | + | - | - | - | - | 1 |
| Zhou 2015 | CS | - | - | + | + | - | - | - | 2 |
| Lee 2015 | CS | - | - | + | - | - | - | - | 1 |
| Pan 2015 | CS | - | - | + | - | - | - | - | 1 |
| Guo 2015 | CS | - | - | + | - | - | - | - | 1 |
| Zhou 2014 | CS | - | - | + | + | - | - | - | 2 |
| Guo 2013 | CS | - | - | + | + | - | + | - | 3 |
| Sun 2018 | CS | - | - | - | - | - | - | + | 1 |
| Jones 2007 | Cohort | - | + | + | + | - | + | - | 4 |
| Jones-Jordan 2011 | Cohort | - | + | - | - | - | + | - | 2 |
| Onal 2007 | Cohort | - | + | - | - | - | - | - | 1 |
| Peckham 1977 | Cohort | - | + | - | - | - | - | - | 1 |
| Saw 2006 | Cohort | - | + | + | - | - | + | - | 3 |
| French 2013 | Cohort | - | - | + | + | - | + | - | 3 |
| Guggenheim 2012 | Cohort | - | - | + | + | - | + | + | 4 |
| Oner 2016 | Cohort | - | - | + | - | - | - | - | 1 |
| Li 2015 | Cohort | - | - | + | - | - | - | - | 1 |
| Jones-Jordan 2012 | Cohort | - | - | + | - | - | - | + | 2 |
| Chen 2016 | Cohort | - | - | - | - | - | - | + | 1 |
| Hsu 2017 | Cohort | - | - | - | - | - | - | + | 1 |
| Ma 2018 | Cohort | - | - | - | - | - | - | + | 1 |
| Saxena 2017 | Cohort | - | - | - | - | - | - | + | 1 |
| Wu 2015 | Cohort | - | - | - | - | - | - | + | 1 |
| Parssinen 1993 | Cohort (nRCT) | - | + | - | - | - | - | - | 1 |
| Saw 2000 | Cohort (nRCT) | - | + | + | - | - | - | - | 2 |
| Wu 2013 | CT | + | - | + | + | - | - | - | 3 |
| Li 2018 | CT | - | - | - | + | - | - | - | 1 |
| Yi 2011 | RCT | + | + | + | + | + | - | - | 5 |
| Wu 2018 | cRCT | + | - | - | + | + | - | + | 4 |
| He 2015 | cRCT | + | + | + | + | + | - | + | 6 |
| Jin 2015 | cRCT | + | - | + | + | + | - | + | 5 |
| Ngo 2014 | cRCT | - | - | - | - | + | - | - | 1 |
